# Supplementary material for: Factors impacting adherence to an exercise-based physical therapy program for individuals with low back pain
Source: PLoS One. 2022 Oct 20;17(10):e0276326. doi: 10.1371/journal.pone.0276326 (PMC9584523; doi:10.1371/journal.pone.0276326)
Supplement: Attachment — Submitted filename: DD22-15309.docx [file pone.0276326.s002.docx]

Thank you for the opportunity to review this important paper. Overall it is an important topic that is often under-examined. However, I have a few major and some minor comments that should be addressed before considering this manuscript for publication.

**Major Comments:**

1) It would be beneficial to define adherence in the introduction. In the introduction and the abstract, it is unclear if adherence has to do with adhering to the rehabilitation program, which includes attending appointments or adhering to the program provided by the rehab experts, such as home exercise programs or general physical activity recommendations. Part of this is the novelty of this research, most research focuses on adherence to HEP and not adherence to attending PT sessions, so the readers can get easily confused.

2) In the introduction, you mention logistic limitations, such as lack of insurance coverage. Thus it is highly recommended that insurance coverage is added to the independent predictor variables included in your model. It is also recommended that lack of geographic accessibility is incorporated into the models as they are the top factors you identified as being contributors to non-adherence.

3) Most of the discussion are the results written in a narrative format. It is recommended that the authors expand on the discussion to add insight into the "so what?"

**Minor Comments:**

Abstract:

1) Your objective is related to predictors of adherence and reasons for non-adherence. But your results are primarily about those who discontinued the program. Recommend combining the first four sentences and expanding on the last one, which relates more to the objectives.

2) The conclusion should relate to your results. I think re-writing your results will help with this.

Introduction:

1) Some more background information about potential factors/expanding on the following: "Logistic limitations such as lack of insurance coverage and accessibility restrictions have been shown to affect trial enrollment and may reduce selection of populations with restricted resources." This would also help justify the reason you picked the four discharge categories on top of page 8.

Methods:

1) line 116-please specify wat clinics, outpatient physical therapy clinics?

2) It would be valuable to justify your independent predictors in the introduction or in the methods. Currently, most of the predictor variables seem random. Furthermore, some of the predictors included in the model per Table 2 are not presented in the methods. Including these in the methods would decrease the appearance of the predictors being at random.

3) Since chart data is used, it would be valuable to have a section on how missingness will be/was handled.

Results:

1) Please add a footnote on abbreviations used in the table.

2) Table 1- I find it hard to believe that Baseline VAS is significant when the 95%CI includes 1. Please double-check these values.

Discussion:

1) I fully believe that this is novel and understudied, but I can't entirely agree with the second statement as a quick google search found the following article and results:

<https://bmcmusculoskeletdisord.biomedcentral.com/articles/10.1186/1471-2474-11-124>

“actors that significantly correlated with adherence included: age (r = 0.7, p < 0.05), initial pain intensity (r = 0.5, p < 0.05), and subjective report of improvement (r = 0.7, p < 0.01). Adherence did not correlate with the type of LBP, patient occupation, experience or nationality of the physiotherapist."

2) I would like to see references or further support regarding the conclusion written on line 259, "suggesting adherence is not affected by insufficient health capacity."

3) The information in paragraph three of the discussion strengthens the reasons why this study should be conducted but doesn't expand on the results. I recommend moving most of this information into the introduction.

4) Throughout the paper, you mention supervised sessions, but this is different from skilled physical therapy sessions. At some point, this needs to be differentiated. Are these patients receiving skilled physical therapy or participating in a supervised exercise program? I say this because many of the references you are using in the discussion are from supervised exercise programs and not skilled physical therapy, and adherence to these are expected to be different.
